# Supplementary material for: HIVprotI: an integrated web based platform for prediction and design of HIV proteins inhibitors
Source: J Cheminform. 2018 Mar 9;10:12. doi: 10.1186/s13321-018-0266-y (PMC5845081; doi:10.1186/s13321-018-0266-y)
Supplement: Supplementary file 2 — Additional file 2. Source code of HIVProtI web server. [file 13321_2018_266_MOESM2_ESM.zip › HIVprotI_Source-code/hivprotI/weka-3-6-6/doc/weka/gui/LookAndFeel.html]

LookAndFeel


---


|  |  |  |
| --- | --- | --- |
| **Overview** \| **Package** \| **Class** \| **Tree** \| **Deprecated** \| **Index** \| **Help** \| **Weka's home** | |  |
| **PREV CLASS**   **NEXT CLASS** | **FRAMES**    **NO FRAMES**     **All Classes** |  |
| SUMMARY: NESTED \| FIELD \| CONSTR \| METHOD | DETAIL: FIELD \| CONSTR \| METHOD |  |


---


## weka.gui Class LookAndFeel

```
java.lang.Object
  weka.gui.LookAndFeel
```

---

``` public class LookAndFeel extends java.lang.Object ```

A little helper class for setting the Look and Feel of the user interface.
Was necessary, since Java 1.5 sometimes crashed the WEKA GUI (e.g. under
Linux/Gnome). Running this class from the commandline will print all
available Look and Feel themes.

**Version:**
:   $Revision: 7059 $

**Author:**
:   FracPete (fracpete at waikato dot ac dot nz)

---

| **Field Summary** | |
| --- | --- |
| `static java.lang.String` | `PROPERTY_FILE`             The name of the properties file |


**Constructor Summary**

`LookAndFeel()`


| **Method Summary** | |
| --- | --- |
| `static java.lang.String[]` | `getInstalledLookAndFeels()`             returns an array with the classnames of all the installed LnFs |
| `static java.lang.String` | `getSystemLookAndFeel()`             returns the system LnF classname |
| `static void` | `main(java.lang.String[] args)`             prints all the available LnFs to stdout |
| `static boolean` | `setLookAndFeel()`             sets the look and feel to the one in the props-file or if not set the default one of the system |
| `static boolean` | `setLookAndFeel(java.lang.String classname)`             sets the look and feel to the specified class |

**Methods inherited from class java.lang.Object**

`equals, getClass, hashCode, notify, notifyAll, toString, wait, wait, wait`

**Field Detail**

### PROPERTY\_FILE

```
public static java.lang.String PROPERTY_FILE
```

:   The name of the properties file


**Constructor Detail**

### LookAndFeel

```
public LookAndFeel()
```


**Method Detail**

### setLookAndFeel

```
public static boolean setLookAndFeel(java.lang.String classname)
```

:   sets the look and feel to the specified class

    :   **Parameters:**: `classname` - the look and feel to use **Returns:**: whether setting was successful

---


### setLookAndFeel

```
public static boolean setLookAndFeel()
```

:   sets the look and feel to the one in the props-file or if not set the
    default one of the system

    :   **Returns:**: whether setting was successful

---


### getSystemLookAndFeel

```
public static java.lang.String getSystemLookAndFeel()
```

:   returns the system LnF classname

    :   **Returns:**: the name of the System LnF class

---


### getInstalledLookAndFeels

```
public static java.lang.String[] getInstalledLookAndFeels()
```

:   returns an array with the classnames of all the installed LnFs

    :   **Returns:**: the installed LnFs

---


### main

```
public static void main(java.lang.String[] args)
```

:   prints all the available LnFs to stdout

    :   **Parameters:**: `args` - the commandline options


---


|  |  |  |
| --- | --- | --- |
| **Overview** \| **Package** \| **Class** \| **Tree** \| **Deprecated** \| **Index** \| **Help** \| **Weka's home** | |  |
| **PREV CLASS**   **NEXT CLASS** | **FRAMES**    **NO FRAMES**     **All Classes** |  |
| SUMMARY: NESTED \| FIELD \| CONSTR \| METHOD | DETAIL: FIELD \| CONSTR \| METHOD |  |


---
